# Yupingfeng Pulvis Regulates the Balance of T Cell Subsets in Asthma Mice

**DOI:** 10.1155/2016/6916353

**Published:** 2016-04-10

**Authors:** Zhigang Wang, Xueting Cai, Zhonghua Pang, Dawei Wang, Juan Ye, Kelei Su, Xiaoyan Sun, Jing Li, Peng Cao, Chunping Hu

**Affiliations:** ^1^Affiliated Hospital of Integrated Traditional Chinese and Western Medicine, Nanjing University of Chinese Medicine, Nanjing, Jiangsu 210028, China; ^2^Laboratory of Cellular and Molecular Biology, Jiangsu Province Academy of Traditional Chinese Medicine, Nanjing, Jiangsu 210028, China

## Abstract

*Background*. Yupingfeng Pulvis (HFBP) had played an active role in many diseases, especially respiratory tract infections. Exploring the possible prevention mechanism of HFBP may provide new ideas in clinical applications for this well-known herbal formula.* Purpose*. To study the possible mechanisms of therapy effect of HFBP on asthma mice via regulating the balance of Tregs and Th17 cells.* Method*. The female BALB/c mice were divided into five groups: control group, model group, prednisone (5.5 mg/kg) group, and 22 g/kg HFBP and 44 g/kg HFBP groups. Ovalbumin was used to make the asthma model of mice; the drug was ig administered daily after atomization for consecutive 15 d. The mice were killed after the last administration. The paraffin-embedded tissue sections of the lungs were stained by H&E. Tregs and Th17 cells in bronchoalveolar lavage fluid were detected by flow cytometry. IL-4, TGF-*β*, and TNF-*α* in the serum were detected by ELISA assay.* Results*. HFBP could alleviate the inflammation in the lung tissue of mice, decrease the proportion of Th17 cells, and increase the proportion of Treg cells in bronchoalveolar lavage fluid. HFBP could decrease IL-4 and TNF-*α* level and increase TGF-*β* level in blood.* Conclusion*. HFBP could treat the asthma through impacting the balance of Th17 cells and Treg cells as well as the levels of related inflammatory cytokines in asthma mice.

## 1. Introduction

Asthma is a common chronic inflammatory disease of the airways characterized by variable and recurring symptoms, reversible airflow obstruction, and bronchospasm. Common symptoms include wheezing, coughing, chest tightness, and shortness of breath. Over the past few decades the world asthma prevalence and mortality have been a rising trend year by year, having impact on social economy and health. The disease is affecting more than 300 million persons all over the world, with approximately 250,000 annual deaths [1], and it is expected that the number of the patients will increase by more than 100 million by 2025 [2].

The pathogenesis of asthma is not yet clear and may be related to genetic, immune, environment, spirit, sex, and other related factors, in which the immunological pathogenesis of asthma has become a current research hot spot. Asthma is classically recognized as the typical Th2 disease, with increased IgE levels and eosinophilic inflammation in the airway. Emerging Th2 cytokines modulate the airway inflammation, which induces airway remodeling [3]. Biological agents, which have specific molecular targets for these Th2 cytokines, are available and clinical trials for asthma are ongoing. However, the relatively simple paradigm has been doubted because of the realization that strategies designed to suppress Th2 function are not effective enough for all patients in the clinical trials. In the future, it is required to understand more details for phenotypes of asthma.

Nowadays, it is known that Th17 cells and Treg cells also modulate asthma. Th17 cells produce IL-17A, IL-17F, and IL-22. These cytokines induce airway inflammation and IL-17A enhances smooth muscle contractility. IL-17 can promote fibroblast cells, epithelial cells, endothelial cells, macrophages, and smooth muscle cells activation, make these cells highly express a variety of proinflammatory factor, such as IL-6 and IL-8, and release granulocyte colony stimulating factor. IL-17 also recruits dendritic cells, T cells, and neutrophils to inflammation section, increasing airway inflammation [4, 5]. CD4^+^CD25^+^Foxp3^+^ regulatory T cells (regulatory T cells, Tregs) are a group which has the function of immune suppression of T cell subgroup. Treg cells produce inhibitory cytokines (TGF-*β* and IL-10) and express membrane molecules (CTLA-4, GITR, etc.) and Foxp3. Studies have found that, in patients with asthma, the specific transcription factor Foxp3 expression reduced, and the inhibition function and the proportion of CD25^hi^ regulatory T cells also reduced [6, 7]. Therefore, studying the differentiation and regulation mechanism of Th17 cell will help us to deepen understanding of the relationship between Th17 cells and the pathogenesis of asthma, as well as the development of new immunosuppressive drugs.

Yupingfeng Pulvis (HFBP) is recorded in Effective Prescriptions Handed Down for Generations written by Yi-lin Wei in Yuan Dynasty. It consists of three commonly used herbs including* Saposhnikovia divaricata* (*Trucz.*)* Schischk.*,* Astragalus membranaceus* (*Fisch.*)* Bunge.,* and* Atractylodes macrocephala Koidz*. Currently, clinical reports about HFBP are increasing. It had played an active role in many diseases, especially treatment of respiratory tract infections. To study the possible mechanisms of therapy effect of HFBP on asthma, asthma model mice were established and the balance of Tregs and Th17 cells was evaluated. Exploring the possible prevention mechanism of HFBP may provide new ideas in clinical applications for this well-known herbal formula.

## 2. Materials and Methods

### 2.1. Animals

Female BALB/c mice were purchased from Shanghai SLRC Laboratory Animal Co., Ltd. (Shanghai, China). Mice were housed under specific pathogen-free conditions and provided with a standard rodent laboratory diet from Shanghai SLRC Laboratory Animal Co., Ltd.

### 2.2. HFBP Preparation

HFBP is composed of three medicinal components:* Saposhnikovia divaricata* (*Trucz.*)* Schischk.*,* Astragalus membranaceus* (*Fisch.*)* Bunge.*, and* Atractylodes macrocephala Koidz* (Table 1). All medicinal plants used to prepare formulas were provided by Jiangsu Province Hospital on Integration of Chinese and Western Medicine (Nanjing, Jiangsu, China). The plant name and part used were shown in Table 1 (the plant name has been checked with http://www.theplantlist.org). All the herbal drugs were authenticated by Professor Song-Lin Li (Jiangsu Province Academy of Traditional Chinese Medicine, Nanjing, China) according to the monographs documented in the Chinese Pharmacopeia (Part I, 2010 Version). Voucher specimens of crude drugs were deposited at the Laboratory of Cellular and Molecular Biology at Jiangsu Province Academy of Traditional Chinese Medicine (Nanjing, China). HFBP extract was prepared according to the following procedure: single crude herb was homogenized with a Waring blender. The powders of three medicinal herbs were mixed in proportion (Table 1) and refluxed with ten volumes of water for 2 h after maceration for 24 h. The filtrates obtained from 2 cycles of the extraction procedure were combined and dried by a vacuum-drier at 60°C and ground. The yield of dried extracts for HFBP was 22% (w/w) of the weight of original herbs.

### 2.3. HPLC Analysis of HFBP

After the centrifugation at 3000 rpm for 5 min, the HFBP supernatant was filtered by membrane filter (0.45 *μ*m) and subjected for HPLC analysis. The experiment was conducted by an Agilent 1200 HPLC instrument (Agilent, USA) equipped with a XTerra@ MS C18 (250 mm × 4.6 mm, 5 *μ*m) column. The mobile phase consisted of 0.1% (v/v) formic acid (A) and acetonitrile (B) at a flow rate of 1.0 mL/min. A gradient program was used as follows: 0 min, 5% B; 10 min, 15% B; 20 min, 20% B; 30 min, 28% B; 40 min, 40% B; 45–50 min, 60% B; 60 min, 70% B; 65–70 min, 95% B. The diode array detector scanned from 200 nm to 400 nm, and the monitor wavelength was set at 250 nm.

### 2.4. The Establishment of Mouse Models of Asthma

We used a protocol slightly modified from that described by McMillan and Lloyd [8]. Briefly, female BALB/c mice were sensitized intraperitoneally with 20 *μ*g ovalbumin (OVA) and 2 mg Al(OH)_3_ on days 1 and 14 (OVA groups, *n* = 40). The blank control group (*n* = 10) was injected with saline at the respective time points. On day 28, OVA groups' mice were random divided into four groups (model group, 22 g/kg HFBP group, 44 g/kg HFBP group, and 5.5 mg/kg prednisone group) (*n* = 10) and inhaled aerosol 1% OVA solution for 30 min for five days. The blank control group was given the respective vehicle aerosol inhalation. HFBP groups with different doses were administrated intragastrically with HFBP every day. 5.5 mg/kg prednisone was administrated intragastrically every day to the positive control group. The blank control group and model group were administrated with equal volume of saline since aerosol inhalation. All groups were administrated for 15 days. Blood was drawn at 24 h after the last intragastric administration to detect cytokines IL-4, TGF-*β*, and TNF-*α*. Bronchoalveolar lavage fluid (BALF) was collected to count the number of Th17 cells and Treg cells. Left upper lung lobes were collected for pathologic histology.

### 2.5. ELISA

The concentrations of cytokines IL-4, TGF-*β*, and TNF-*α* in the blood from all groups were measured using a commercially available ELISA kit (Nanjing Jiancheng Bioengineering Institute, China). ELISA assay was performed according to the manufacturer's instructions of the ELISA kits.

### 2.6. Flow Cytometric Analysis

For detecting the percentage of Th17 cells, cells in BALF were collected and stimulated with 10 ng/mL phorbol 12-myristate 13-acetate (PMA) (Sigma-Aldrich, USA) and 10 ng/mL brefeldin A (BFA) (Sigma-Aldrich, USA) in RPMI-1640 medium (Invitrogen, USA) in 96-well plates. After being stimulated for 5 h (37°C, 5% CO_2_), the cells were collected and washed once with PBS. The cells were then incubated with CD4-FITC antibody at 4°C for 30 minutes. Next, the cells were fixed and permeabilized and stained with anti-human IL-17-PE antibody at 37°C for 30 minutes. For detecting the percentage of Treg cells, the cells were washed in PBS. Then, the cells were stained with CD4-FITC and CD25-APC antibodies at 4°C for 30 minutes. Then, the cells were incubated with Foxp3-PE antibody after fixation and permeabilization according to the manufacturer's instruction. All stained cells were analyzed by flow cytometer (Guava 6HT, Merck-Millipore, USA). The data were analyzed using the software Guava 2.5.

### 2.7. Immunohistochemistry Assay

Lung tissue samples of each group were cut into sections of approximately 0.5 cm^2^ sizes and fixed in 10% formalin for at least 48 hours. The fixed samples were placed in plastic cassettes and dehydrated using an automated tissue processor. The processed tissues were embedded in paraffin wax (Leica, Germany) and the blocks trimmed and sectioned to about 5 × 5 × 4 *μ*m size using a microtome. The tissue sections were mounted on glass slides using a hot plate and subsequently treated in order with 100, 90, and 70% ethanol for two minutes each. Finally, the sections were rinsed with tap water and stained with Harris's haematoxylin and eosin for light microscopy.

### 2.8. Statistical Evaluation

All results shown represent the Mean ± SD from triplicate experiments performed in a parallel manner unless otherwise indicated. Statistical analyses were performed using an unpaired, two-tailed Student's *t*-test.

## 3. Results

### 3.1. HPLC Fingerprint of HFBP Extracts

**Figure 1:**
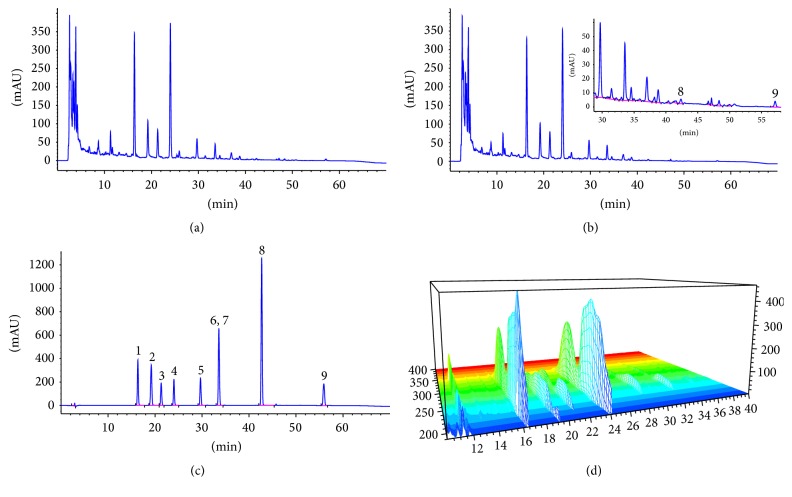
HPLC fingerprint of HFBP extracts and reference compounds. (a) HFB extract (batch I), (b) HFB extract (batch II), (c) reference compounds, (d) 3D-HPLC fingerprint of HFBP extract (batch I). 1, prim-O-glucosylcimifugin; 2, calycosin-7-O-*β*-D-glucoside; 3, macrotin; 4, 5-O-methylvisammioside; 5, ononin; 6, calycosin; 7, sec-O-glucosylhamaudol; 8, formononetin; 9, atractylenolide I.

HPLC analysis was conducted to confirm the biological composition of HFBP extract, resulting in the identification of 9 chemical components with reference standards (Figure 1). Since HFBP is a polyherbal formulation with complicated composition, two batches of HFBP extracts were thus prepared under the same condition and analyzed by HPLC. The results showed that all the main chromatographic peaks detected in batch I coincided with batch II, demonstrating a good reproducibility of HFBP chemical composition. Figure 1 shows the HPLC fingerprint of two batches of HFBP extracts and 3D-HPLC chromatogram of batch I HFBP extracts. In order to evaluate the quality of HFBP extracts, an external standard method was applied to quantitatively analyze five major compounds (prim-O-glucosylcimifugin, calycosin-7-O-*β*-D-glucoside, macrotin, 5-O-methylvisammioside, and ononin) in the HFBP samples. The external standard method was validated in terms of linearity, precision, accuracy, and stability. The quantitative results are presented in Table 2.

### 3.2. HFBP Impact on Serum Inflammatory Factors in Asthma Mice

**Figure 2:**
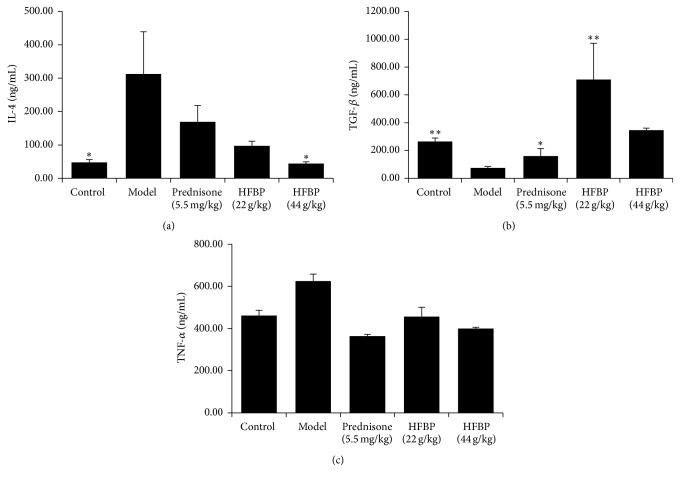
Detection of levels of IL-4 (a), TGF-*β* (b), and TNF-*α* (c) in serum of mice by ELISA (Mean ± SD, *n* = 10). ^*∗*^ *P* < 0.05 and  ^*∗∗*^ *P* < 0.01 versus model group.

Experiments were made to determine the serum inflammatory cytokines IL-4, TGF-*β*, and TNF-*α*; as shown in Figure 2, IL-4 and TNF-*α* increased in model group; two HFBP groups could significantly reduce serum IL-4 and TNF-*α* levels, so did prednisone group. TGF-*β* as a suppression of inflammation factor reduced in the model group; prednisone can raise TGF-*β* level; by contrast, HFBP groups could increase the level of the serum content of TGF-*β* more than prednisone group.

### 3.3. The Percentages of Th17 and Treg Cells in BALF in Five Groups

**Figure 3:**
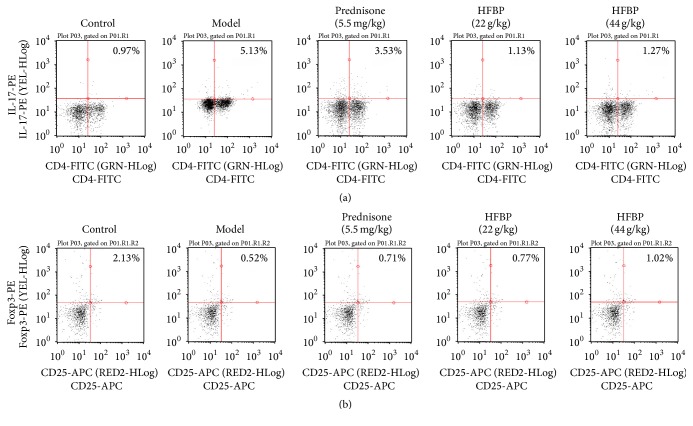
Detection for proportion of Th17 cells (a) and Treg cells (b) in mice by flow cytometry (Mean ± SD, *n* = 10). ^*∗*^ *P* < 0.05 and  ^*∗∗*^ *P* < 0.01 versus model group.

Flow cytometry results showed that the percentage of Th17 cells in model group significantly enhanced compared with blank control group, and the proportion of Treg cells decreased obviously. Positive medicine prednisone could reduce Th17 cells and increase Treg cells. Both HFBP groups could reduce the percentage of Th17 cells and increase the proportion of Treg cells, and the effect of HFBP was better than prednisone (Figures 3(a) and 3(b)).

### 3.4. Effect of HFBP on Lung Tissue Pathology in Asthma Mice

**Figure 4:**
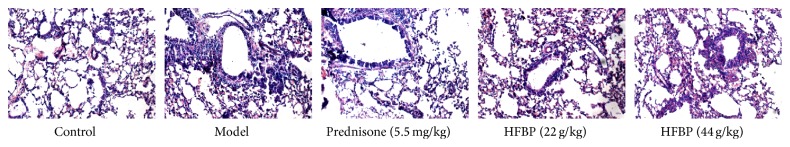
H&E staining of lung of asthma mice of each group (optical microscopy, ×200).

H&E staining of lung tissue showed that there were no obvious pathological changes of lung tissue observed in the blank control group mice. In the model group mice, a large number of inflammatory cells infiltrated bronchial wall of lung tissue. The prednisone group could improve the lung tissue of the inflammatory cells invasion; both high and low dose HFBP groups could improve the bronchial inflammation situation; lung damage had a greater degree of recovery after HFBP treatment, showing that HFBP had a good treatment effect, as shown in Figure 4.

## 4. Discussion

Asthma treatment goal is to reduce attack frequency, improve respiratory function and life quality, control acute onset, prevent deterioration, and avoid death. At present the treatment of asthma is mainly western medicine; traditional Chinese medicine (TCM) is used as complementary therapies. Western medicine treatment of asthma is mainly with anti-inflammatory drugs, complementary with spasmolytic agents and apophlegmatisant. Western medicine has achieved very good curative effect in the control of asthma symptoms but still cannot reduce the recurrence rate of asthma and has the side effects due to long-term use of these drugs. Traditional Chinese medicine has a long history in treating asthma, and has rich pharmaceutical with low cost and small side effects. Traditional Chinese medicine blocked a chain reaction of inflammation, in particular, to improve the microenvironment to eliminate airway chronic inflammation in the airway, prevent the occurrence of airway remodeling, and regulate the immune system by changing the immune gene expression, reduce airway hyperresponsiveness, stabilize environment within the body, and enhance the adaptive adjustment ability. TCM gives full play to the advantages of the overall treatment.

The ingredients of HFBP are fewer but better, only three components,* radix astragali*,* radix saposhnikoviae,* and* rhizoma atractylodis macrocephalae*.* Radix astragali* is especially suitable for the treatment of deficiency and night sweat people, which is the main drug of the formulas.* Rhizoma atractylodis macrocephalae* is the adjuvant drug of the formulas.* Radix saposhnikoviae*, called “Pinfeng,” can release exterior and dispel wind. In recent years, more and more pharmacological effects of HFBP have been revealed for its “replenishing qi and consolidating exterior” pharmacodynamics basis. Previous studies have shown that HFBP has good immunity effect on allergic diseases such as asthma, allergic rhinitis, and allergic conjunctivitis [9–11]. Its pharmacological mechanism may be through promoting Th1 cells and the expression of IFN-*γ*, inhibition of Th2 cells, and the secretion of cytokines, thus improving the Th1/Th2 ratio and the state of inflammation of the respiratory tract [9]. Th17 cells and Treg cells have obvious change in bronchial asthma patients. Higher levels of Th17 cells raise the inflammation of lung tissue; lower level of Treg cells reduces the inhibition effect of inflammatory cells and inflammatory factor, which can promote the occurrence of asthma disease [12, 13]. HFBP has a good immunity effect on allergic diseases, yet there is no evidence about its relationship with the balance of Th17 cells and Treg cells.

Therefore, we designed the experiment to investigate the relationship between Th17 cells differentiation mechanism and the onset of asthma and at the same time observe Th17 related cytokines expression with HFBP treatment in acute asthma mice. The results showed that HFBP could reduce asthma mice lung tissue bronchioles and perivascular inflammatory cell infiltration, improve the state of airway inflammation, and reduce mucus secretion. ELISA assay was used to detect IL-4, TGF-*β*, and TNF-*α* level in the blood of mice. According to the results, IL-4 and TNF-*α* of asthma mice were increased, compared with the blank control group (*P* < 0.05), suggesting that IL-4 and TNF-*α* participate in the onset of asthma. After treating with prednisone or HFBP, IL-4 and TNF-*α* level significantly reduced (*P* < 0.05), suggesting that HFBP could inhibit IL-4 and TNF-*α* expression in the blood of asthma mice. TGF-*β* as a suppression of inflammation factor reduced in the model group; after treatment with prednisone or HFBP, TGF-*β* level increased obviously. Flow cytometry analysis showed that HFBP could decrease the proportion of Th17 cells and increase the proportion of Treg cells in bronchoalveolar lavage fluid, which indicated that HFBP could treat the asthma through impacting the balance of Th17 cells and Treg cells as well as the levels of related inflammatory cytokines in mice.

Differentiation of naïve T cells into effector cells is required for optimal protection against different classes of microbial pathogen and for the development of immune memory. Recent findings have revealed important roles for the Notch signaling pathway in T cell differentiation into all known effector subsets, including Th1, Th2, and Tregs [14]. Inhibiting Notch signaling has been shown to block Th2 polarization by preventing Notch mediated upregulation of GATA-3. Zhou found that Astragalus injection exerted protective effects on bleomycin-induced pulmonary fibrosis via downregulating Jagged1/Notch1 in lung [15]. Atractylenolide I, one of the main naturally occurring compounds of* Atractylodes macrocephala Koidz.*, had the effect of reduction of expressions of Notch1, Jagged1, and downstream protein Hes1, Hey1 of Notch pathway [16]. Above all, we doubted that HFBP may impact the balance of Th17 cells and Treg cells through Notch signaling pathway. Of course, further experiments needed to be done.

## Figures and Tables

**Table 1:** The composition of HFBP.

Species	Chinese name	Plant part	Origin	Grams, g	%
*Saposhnikovia divaricata (Trucz*.*) Schischk*.	Fangfeng	Root	Hebei, China	30	25.0
*Astragalus membranaceus (Fisch*.*) Bunge*.	Huangqi	Root	Shanxi, China	30	25.0
*Atractylodes macrocephala Koidz*.	Baizhu	Rhizoma	Jiangsu, China	60	50.0

Total amount				120	100.0

**Table 2:** The contents of five major compounds in HFBP by HPLC analysis.

Peak number	*t*_*R*_ (min)	Compound name	Linearity		Sensitivity (*µ*g/mL)		Precision (RSD%, *n* = 6)		Stability (RSD%, *n* = 3)	Content (%)
			Equation	*R* ^2^	LOD	LOQ	Intra-day	Inter-day		
1	16.37	Prim-O-glucosylcimifugin	*y* = 40565*x* + 149.55	0.9998	0.03	0.05	0.24	1.38	0.40	0.202
2	19.23	Calycosin-7-O-*β*-D-glucoside	*y* = 73499*x* + 64.019	0.9997	0.027	0.055	0.26	1.58	0.51	0.043
3	21.33	Macrotin	*y* = 60171*x* + 50.145	0.9997	0.047	0.023	0.16	1.59	0.56	0.035
4	24.03	4′-O-*β*-D-Glucosyl-5-O-methylvisamminol	*y* = 45658*x* + 179.85	0.9996	0.031	0.051	0.17	1.33	0.18	0.221
5	29.69	Ononin	*y* = 76593*x* + 60.616	0.9997			0.20	1.40	0.48	0.021

## Abbreviations

HFBP: Yupingfeng Pulvis
TCM: Traditional Chinese medicine
Tregs: Regulatory T cells
OVA: Ovalbumin
BALF: Bronchoalveolar lavage fluid.
